# Supplementary material for: Activity dependent feedback inhibition may maintain head direction signals in mouse presubiculum
Source: Nat Commun. 2017 Jul 20;8:16032. doi: 10.1038/ncomms16032 (PMC5524997; doi:10.1038/ncomms16032)
Supplement: Supplementary Information [file ncomms16032-s1.pdf]

Title of file for HTML: Supplementary Information
Description: Supplementary Figures and Supplementary Tables

Title of file for HTML: Peer Review File
Description:

	MC			Significant difference	PC		
	mean	sd	n		mean	sd	n
Membrane Potential (mV)	-51.1	6.9	80	**** p < 0.0001	-76.3	5.9	87
Time constant (ms)	37.1	17.6	84	**** p < 0.0001	22.5	12	90
R_{in} (MOhm)	343	129	74	**** p < 0.0001	251	112	85
Sag ratio	1.22	0.01	72	**** p < 0.0001	1.05	0.02	74
Threshold current (pA)	51.5	38.9	64	**** p < 0.0001	92.3	50.4	65
I-O initial gain (Hz/pA)	0.845	0.040	64	**** p < 0.0001	0.373	0.127	65
Firing frequency @ 2x Threshold current (Hz)	37.5	26.0	64	ns p = 0.5892	32.25	11.51	64
AP rising amplitude (mV)	79.8	6.2	86	ns p = 0.1069	81.69	5.86	95
AP half duration (ms)	0.293	0.039	86	**** p < 0.0001	0.578	0.103	95
AP rising phase speed (V/s)	565	87	86	**** p < 0.0001	508	86	95
AP falling phase speed (V/s)	-319	59	86	**** p < 0.0001	-131	19	95

Supplementary Table 1, related to Figure 1. Electrophysiological properties of presubicular pyramidal cells and Martinotti interneurons. Values are given as mean ± sd. The column "significant difference" indicates the p-values for a two-tailed Mann Whitney U test comparing the two cell populations.

a **Injection site**

mouse n°	AD	AV	LD	RT	LHb
#616	++++	++++	+	+	+
#617	++++	++++	++	-/+	-
#621	++++	+++	+	-/+	+
#622	+++++	+++++	++	+	+/-
#623	++++	+++	++	+	+

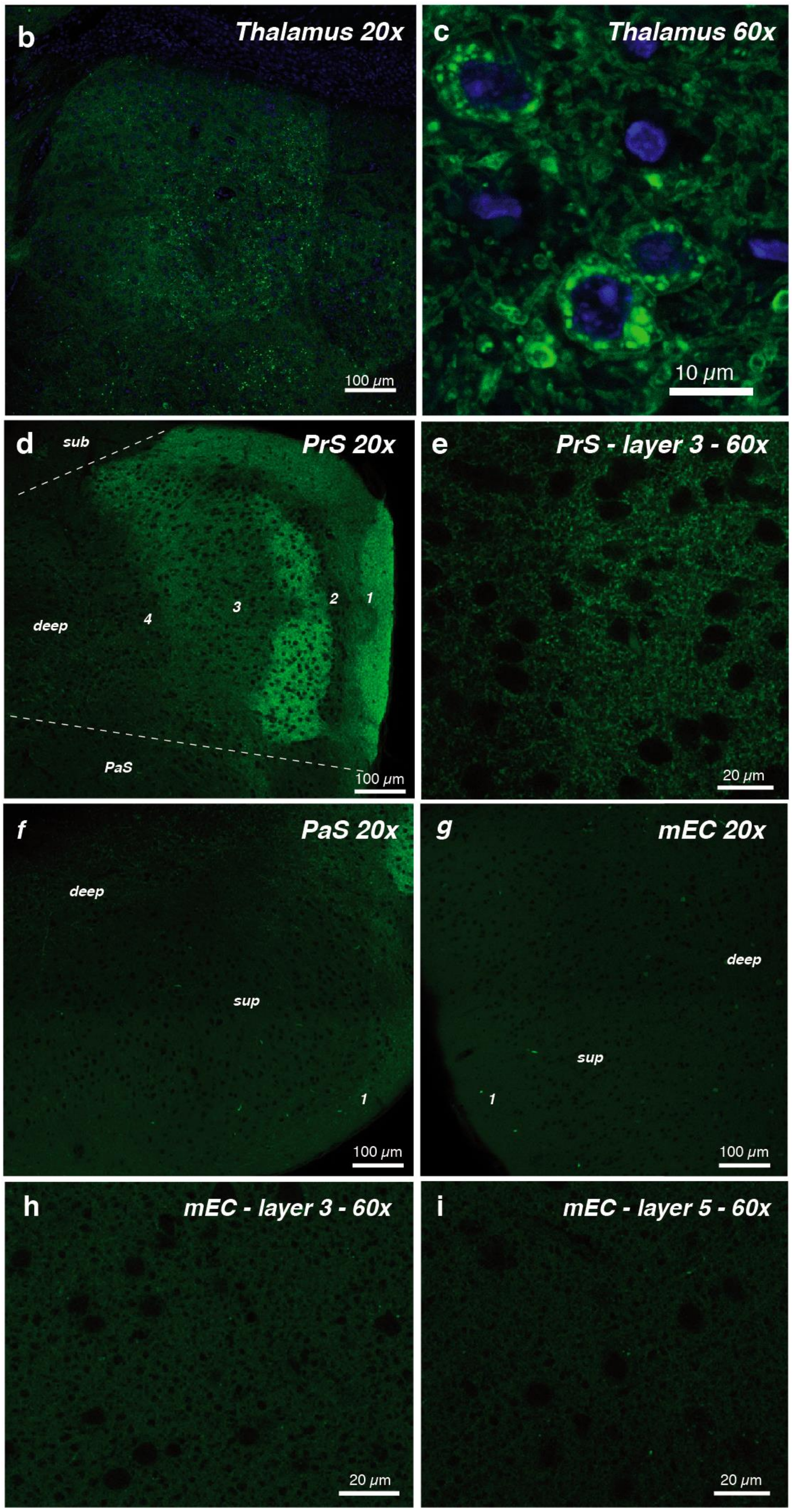

Supplementary Figure 1. related to Figure 2. Virus injection site in the thalamus and thalamic fibers in higher magnification images of the parahippocampal area.
(a) injection site specificity. Images showing virus injection site in the thalamus at **(b)** 20x and **(c)** 60x magnification. **(d)** 20x images showing presubiculum **(f)** parasubiculum and **(g)** entorhinal cortex. We also provide a 60x image of layer 3 of presubiculum **(e)** and of entorhinal cortex layer 3 **(h)** and 5 **(i).**

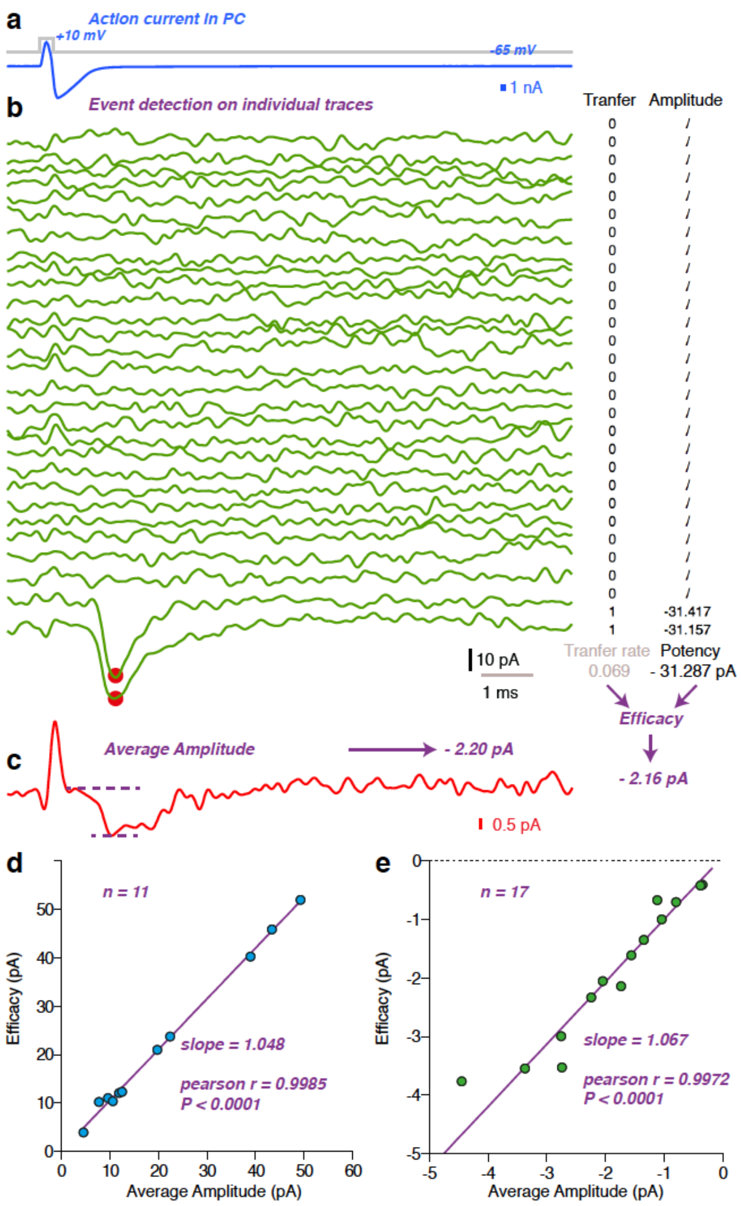

Supplementary Figure 2, related to Figure 3. Two reliable methods to determine synaptic strength: measures of average amplitude or trial-by-trial analysis to calculate efficacy give equivalent results.

(a) A single action current was evoked in a pyramidal cell with a very high (-65 to +10 mV) and short (0.2 ms) voltage pulse.

(b) Martinotti cell was recorded in voltage clamp mode at -65 mV. The postsynaptic currents in the Martinotti cell (green traces) following the stimulation are shown for twenty-nine trials. EPSCs were evoked within a monosynaptic time window (0.5 to 3 ms) only in two trials. Transfer (1 when an EPSC is detected and 0 otherwise) and amplitude of events (nothing when transfer equals 0) is indicated on the right. The mean transfer rate (0.069), potency (-31.287) and the calculated efficacy (-2.16 pA) are indicated.

(c) The average current response of the Martinotti cell is shown in red and its maximum downward amplitude of – 2.20 pA was determined within the monosynaptic window. The upward deflection at the beginning is a stimulation artifact. Note the similarity between trial-by-trial calculation of efficacy and the average trace amplitude.

(d-e) These two methods were applied for n = 11 MC-to-PC synapses **(d)** and n = 17 PC-to-MC synapses **(e)** to test their agreement. Efficacy and Average amplitude were highly correlated for both synapses.

N = 8	Efficacy (pA)						
	10 Hz	10 Hz	10 Hz	30 Hz	30 Hz	30 Hz	late
	early	late	late/early	early	late	late/early	30Hz/10Hz
MCtoPC#1	5.95	6.55	1.1	4.50	5.44	1.2	0.8
MCtoPC#2	9.45	12.31	1.3	9.33	11.54	1.2	0.9
MCtoPC#3	10.13	13.01	1.3	10.20	13.43	1.3	1.0
MCtoPC#4	11.73	9.60	0.8	9.94	8.01	0.8	0.8
MCtoPC#5	15.49	19.40	1.3	16.33	17.21	1.1	0.9
MCtoPC#6	22.43	22.64	1.0	21.02	18.65	0.9	0.8
MCtoPC#7	24.13	18.71	0.8	19.51	19.02	1.0	1.0
MCtoPC#8	36.50	36.40	1.0	38.22	30.48	0.8	0.8
**Mean**	**16.97**	**17.33**	**1.1**	**16.13**	**15.47**	**1.0**	**0.9**
**sd**	**10.12**	**9.40**	**0.2**	**10.54**	**7.81**	**0.2**	**0.1**
**sem**	**3.58**	**3.32**	**0.1**	**3.73**	**2.76**	**0.1**	**0.0**
N = 8	Transfer rate						
	10 Hz	10 Hz	10 Hz	30 Hz	30 Hz	30 Hz	late
	early	late	late/early	early	late	late/early	30Hz/10Hz
MCtoPC#1	0.60	0.59	1.0	0.49	0.51	1.0	0.9
MCtoPC#2	0.67	0.77	1.1	0.67	0.67	1.0	0.9
MCtoPC#3	0.66	0.74	1.1	0.74	0.80	1.1	1.1
MCtoPC#4	0.75	0.69	0.9	0.71	0.60	0.8	0.9
MCtoPC#5	0.75	0.86	1.1	0.77	0.82	1.1	0.9
MCtoPC#6	0.96	0.96	1.0	0.97	0.93	1.0	1.0
MCtoPC#7	0.88	0.80	0.9	0.84	0.79	0.9	1.0
MCtoPC#8	0.97	0.93	1.0	0.93	0.97	1.0	1.0
**Mean**	**0.78**	**0.79**	**1.0**	**0.77**	**0.76**	**1.0**	**1.0**
**sd**	**0.14**	**0.12**	**0.1**	**0.15**	**0.16**	**0.1**	**0.1**
**sem**	**0.05**	**0.04**	**0.0**	**0.05**	**0.06**	**0.0**	**0.0**
N = 8	Potency (pA)						
	10 Hz	10 Hz	10 Hz	30 Hz	30 Hz	30 Hz	late
	early	late	late/early	early	late	late/early	30Hz/10Hz
MCtoPC#1	9.94	11.19	1.1	9.24	10.66	1.2	1.0
MCtoPC#2	13.94	16.31	1.2	13.64	18.10	1.3	1.1
MCtoPC#3	15.29	17.55	1.1	13.66	16.69	1.2	1.0
MCtoPC#4	15.74	13.94	0.9	13.98	13.28	0.9	1.0
MCtoPC#5	20.45	22.55	1.1	20.40	21.05	1.0	0.9
MCtoPC#6	23.54	23.61	1.0	21.68	20.07	0.9	0.8
MCtoPC#7	27.60	23.31	0.8	23.09	23.86	1.0	1.0
MCtoPC#8	37.58	39.23	1.0	40.92	31.42	0.8	0.8
**Mean**	**20.51**	**20.96**	**1.0**	**19.58**	**19.39**	**1.1**	**0.9**
**sd**	**8.91**	**8.67**	**0.1**	**9.86**	**6.44**	**0.2**	**0.1**
**sem**	**3.15**	**3.07**	**0.0**	**3.49**	**2.28**	**0.1**	**0.0**

Supplementary Table 2, related to figure 4. Transmission at the Martinotti cell to pyramidal cell synapses.

N = 9	Efficacy (pA)						
	10 Hz	10 Hz	10 Hz	30 Hz	30 Hz	30 Hz	late
	early	late	late/early	early	late	late/early	30Hz/10Hz
PCtoMC#1	0.00	0.00	+*inf*	1.09	7.54	6.9	+*inf*
PCtoMC#2	0.00	3.90	+*inf*	2.99	6.58	2.2	1.7
PCtoMC#3	0.14	2.12	15.1	1.22	10.92	9.0	5.2
PCtoMC#4	1.46	5.75	3.9	5.63	29.53	5.2	5.1
PCtoMC#5	2.54	4.39	1.7	4.58	7.17	1.6	1.6
PCtoMC#6	2.55	2.13	0.8	4.67	13.31	2.9	6.2
PCtoMC#7	4.35	3.42	0.8	4.67	8.94	1.9	2.6
PCtoMC#8	5.22	7.09	1.4	4.93	18.52	3.8	2.6
PCtoMC#9	9.00	29.78	3.3	9.90	53.52	5.4	1.8
**Mean**	**2.81**	**6.51**	**3.9**	**4.41**	**17.34**	**4.3**	**3.4**
**sd**	**2.99**	**8.97**	**5.1**	**2.63**	**15.42**	**2.5**	**1.9**
**sem**	**1.00**	**2.99**	**1.9**	**0.88**	**5.14**	**0.8**	**0.7**
N = 9	Transfer rate						
	10 Hz	10 Hz	10 Hz	30 Hz	30 Hz	30 Hz	late
	early	late	late/early	early	late	late/early	30Hz/10Hz
PCtoMC#1	0.00	0.00	+*inf*	0.03	0.20	6.67	+*inf*
PCtoMC#2	0.00	0.16	+*inf*	0.10	0.17	1.70	1.06
PCtoMC#3	0.02	0.16	8.00	0.09	0.60	6.67	3.75
PCtoMC#4	0.08	0.18	2.25	0.14	0.64	4.57	3.56
PCtoMC#5	0.16	0.29	1.81	0.36	0.49	1.36	1.69
PCtoMC#6	0.17	0.30	1.76	0.20	0.60	3.00	2.00
PCtoMC#7	0.27	0.18	0.67	0.31	0.44	1.42	2.44
PCtoMC#8	0.26	0.48	1.85	0.28	0.72	2.57	1.50
PCtoMC#9	0.32	0.66	2.06	0.36	0.96	2.67	1.45
**Mean**	**0.14**	**0.27**	**2.63**	**0.21**	**0.54**	**3.40**	**2.18**
**sd**	**0.12**	**0.20**	**2.42**	**0.12**	**0.25**	**2.09**	**1.00**
**sem**	**0.04**	**0.07**	**0.92**	**0.04**	**0.08**	**0.70**	**0.35**
N = 9	Potency (pA)						
	10 Hz	10 Hz	10 Hz	30 Hz	30 Hz	30 Hz	late
	early	late	late/early	early	late	late/early	30Hz/10Hz
PCtoMC#1	/	/	/	38.08	38.04	1.00	+*inf*
PCtoMC#2	/	27.56	+*inf*	29.63	41.16	1.39	1.49
PCtoMC#3	6.40	14.24	2.23	15.11	18.46	1.22	1.30
PCtoMC#4	18.24	29.84	1.64	43.64	46.11	1.06	1.55
PCtoMC#5	14.82	15.32	1.03	12.19	15.40	1.26	1.01
PCtoMC#6	15.31	9.62	0.63	23.80	22.02	0.93	2.29
PCtoMC#7	16.60	19.02	1.15	13.26	19.71	1.49	1.04
PCtoMC#8	22.24	14.50	0.65	16.48	26.00	1.58	1.79
PCtoMC#9	27.38	45.44	1.66	24.73	56.06	2.27	1.23
**Mean**	**17.28**	**21.94**	**1.12**	**24.10**	**31.44**	**1.36**	**1.46**
**sd**	**6.54**	**11.74**	**0.71**	**11.22**	**14.32**	**0.41**	**0.43**
**sem**	**2.47**	**4.15**	**0.25**	**3.74**	**4.77**	**0.14**	**0.15**

Supplementary Table 3, related to figure 5. Transmission at the pyramidal cell to Martinotti cell synapses.

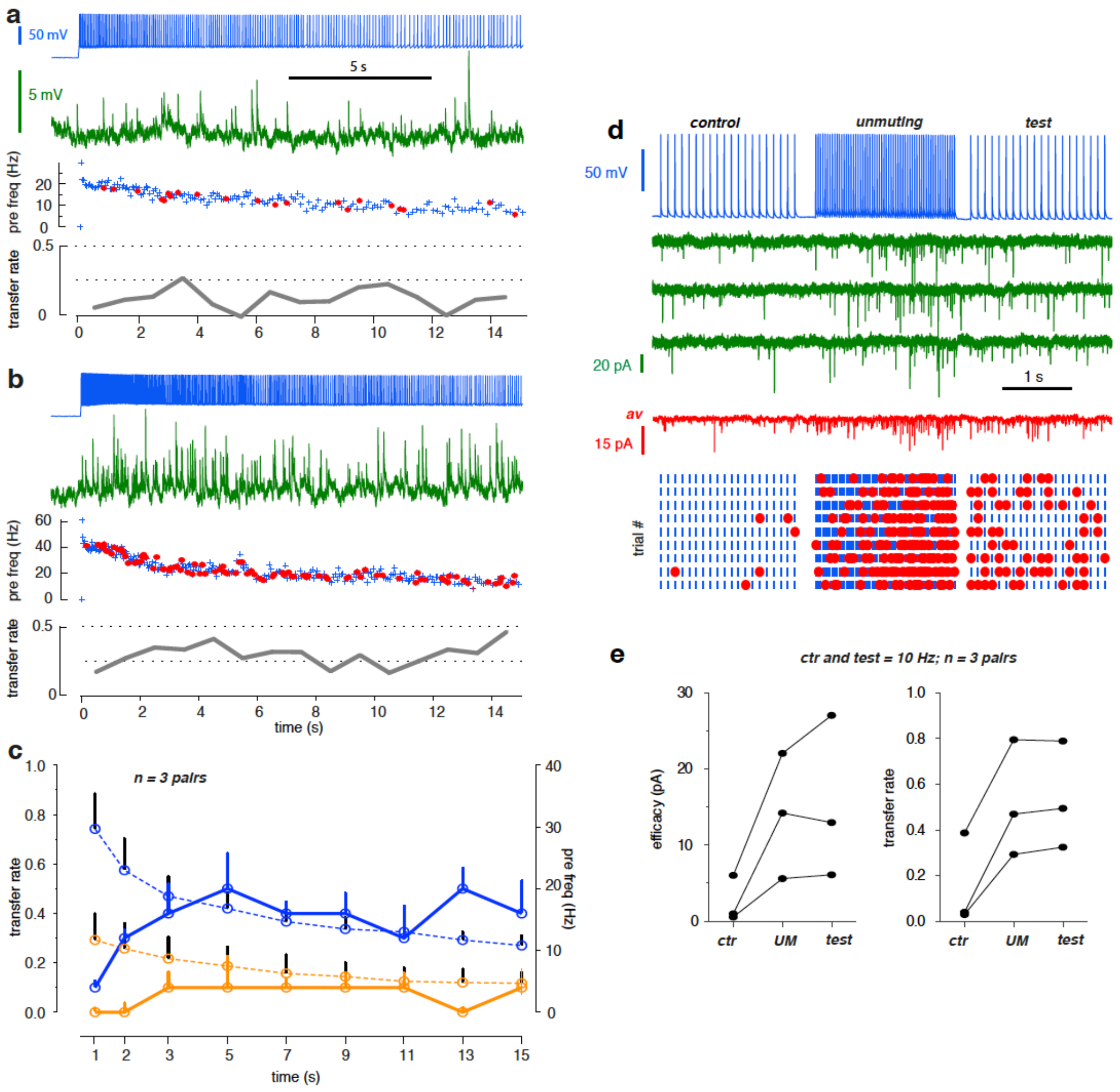

Supplementary Figure 3, related to figure 6. Moderate firing can maintain synaptic un-muting following initial high frequency firing.

(**a,b,c**) Maintained current injections were applied to induce maintained firing in a presynaptic PC while a MC was recorded in current clamp mode. The top graphs show the presynaptic instantaneous frequency over time (blue +). Red dots indicate monosynaptic EPSPs in the postsynaptic MC. The lower graphs show transfer rate over time.

(a) Presynaptic firing frequency adapted from 50 to 20 Hz. The initial 50 Hz spiking activity un-muted the synaptic connection and synaptic transfer remained enhanced (0.2 - 0.5) even as presynaptic firing frequency progressively slowed.

(b) Presynaptic firing frequency adapted from 20 Hz to 10 Hz over several seconds. The initial 20 Hz frequency did not enhance transfer rate, and transfer remained low (0 – 0.25).

(c) For initially high firing frequencies (blue broken line), synapses were un-blocked and transfer rates (blue line) stayed high even as presynaptic firing frequencies progressively decreased. For initially low firing frequency stimulation (orange broken line), synapses remained un-efficient with low transfer rates (orange line). Summary data from n = 3 pairs, mean ± SEM.

(d) PC-stimulation (blue) and MC-EPSCs recorded at -65 mV. Three trials (green) and average in red (low pass filtered at 1 Hz). Below, raster plots of synaptic transfer. The PC was stimulated with a control train at 10 Hz, followed by an unmuting train at 30 Hz and then a test stimulus at 10 Hz. The raster plot shows a low synaptic efficacy during the control 10 Hz train, unmuting and facilitation during the 30 Hz train followed by enhanced transfer during the subsequent 10 Hz test train.
(e) Summary data from n = 3 pairs where synaptic unmuting was successful and synaptic efficacy and transfer rate had doubled at the end of the 30 Hz spike train.

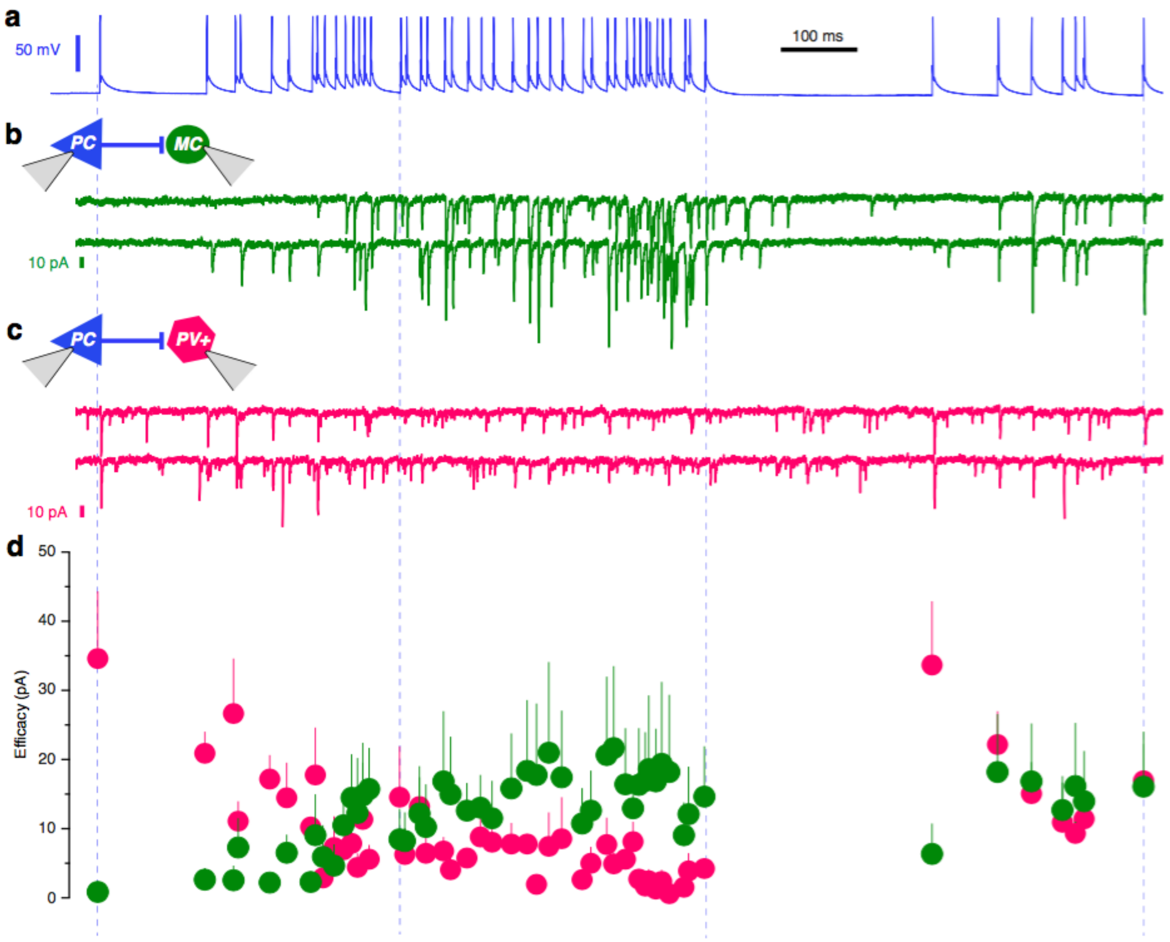

Supplementary Figure 4, related to figure 7. Synaptic responses to HD spike train in Martinotti vs parvalbumin (PV) expressing cells.

(a) Head direction firing pattern injected in a pyramidal neuron.

(b) The recordings from a postsynaptically connected Martinotti cell show EPSCs in response to the spike train, with facilitating dynamics (shown are two consecutive trials).

(c) The recording from a postsynaptically connected parvalbumin positive fast-spiking neuron also show EPSCs in response to the same spike train, but with depressing dynamics (shown are two consecutive trials).

(d) Summary graph of the average efficacy of evoked EPSCs for the Martinotti cells (in green; n = 5 pairs) and the average efficacy of evoked EPSCs in parvalbumin positive cells (in pink; n = 5 pairs). Note how the first spike evoked the largest response in PV cells, while Martinotti neurons didn't respond to the first spike.

N_{Pyr}	500
r	0.25
$\bar{w}^{OUT}$	1
w^{OUT}	0.4
w^{IN}	35
t_E	22 ms
t_I	37 ms
t_N	5 ms
k_1	100
k_2	0.2
b_1	0.00025
b_2	0.5
n	0.1
s	0.01
b	0.4
k	28°
L	0.6668

Supplementary Table 4. Table of model network parameters.

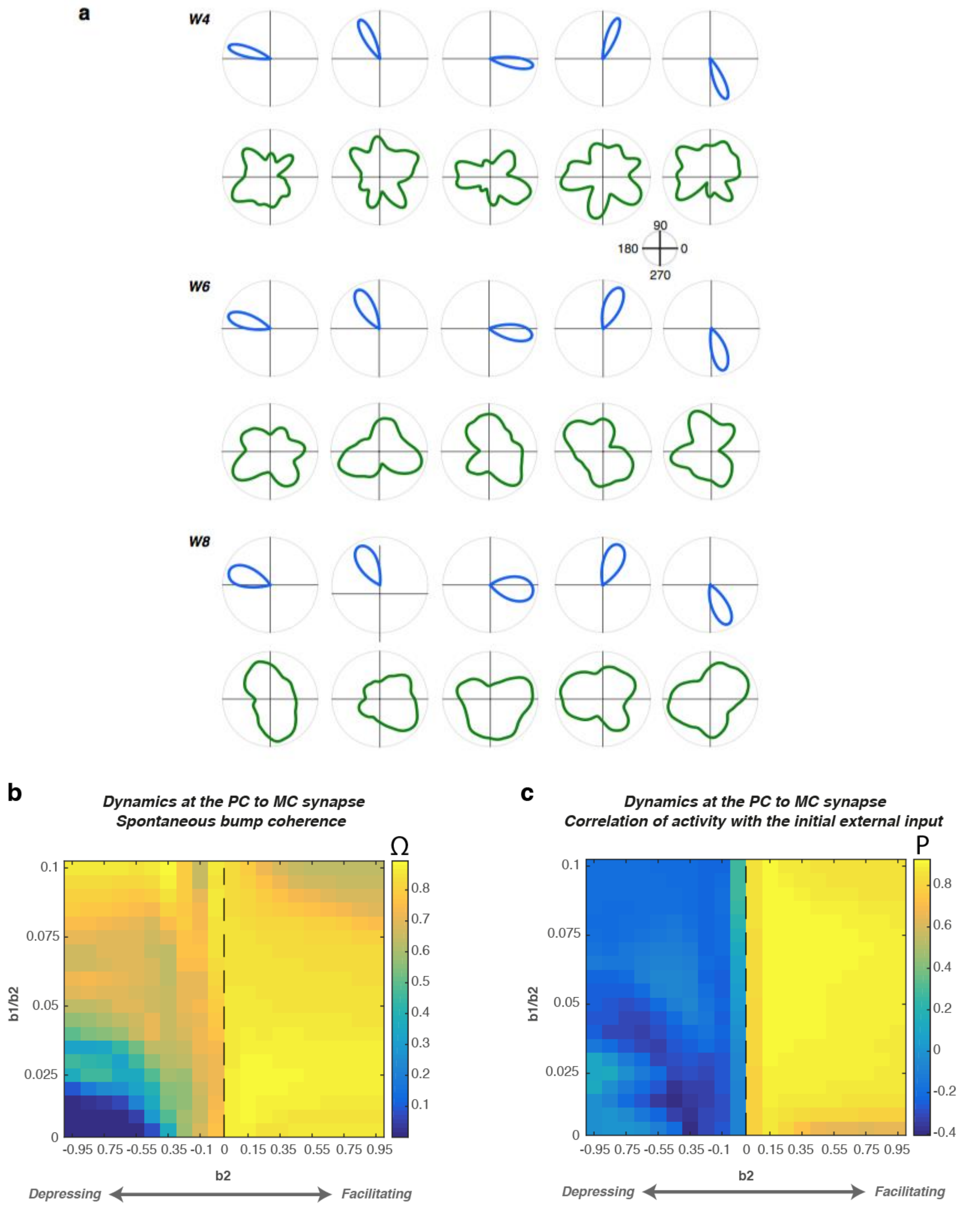

Supplementary Figure 5, related to figure 9.

(a) Examples of tuning curves from running the network simulation. A series of 5 examples for Pyramidal cell polar plots (blue) and Martinotti cell polar plots (green) for different values of the range α of the inhibition suppression (W4 is $\alpha/N=0.04$, W6 is $\alpha/N=0.06$ and W8 is $\alpha/N=0.08$). The larger α, the broader the head directional tuning of pyramidal cells. Martinotti cells are not directionally tuned.

(b,c) How do dynamical properties of excitatory synapses affect network activity? This was examined by changing parameters b1 and b2. b2 > 0 models the potentiation of synaptic strength for repeated pyramidal cell firing (facilitating dynamics), whereas b2 < 0 implies a decrease of synaptic strength

after an initial stimulus (depressing dynamics). The absolute value of b2 determines the strength of the modulation. b1 controls the decay or recovery speed of facilitation or depression; higher values of b1 lead to more persistent modulation.

(b) Coherence of the network activity as a function of the dynamical parameters of the excitatory synapses. The ability of the network to form a coherent bump of activity Ω was measured as the directional tuning of the network following a transient, directionally selective external input. High scores (yellow) correspond to persistent directional coherence in the network activity profile, and are more readily achieved for facilitating dynamics than for depressing dynamics.

(c) Correlation P of the directional activity profile of the network with the initial activity profile. High scores reflect the ability of the network to maintain the directional tuning initially imposed from an external source, and can only be obtained for facilitating synaptic dynamics.
